# ESR Essentials: Gynaecological causes of acute pelvic pain in women: a primer for emergent evaluation—practice recommendations by the European Society of Emergency Radiology

**DOI:** 10.1007/s00330-025-11539-8

**Published:** 2025-05-21

**Authors:** Elizabeth A. Dick, Ana Blanco, Marcela De La Hoz Polo, Raffaella Basilico

**Affiliations:** 1 Imperial College NHS Trust, London, UK; 2 Imperial College, London, UK; 3 Emergency Section, Department of Radiology, Hospital Universitario Morales Meseguer, Murcia, Spain; 4 Everlight Radiology, London, UK; 5 Raffaella Basilico, Department of Medical, Oral and Biotechnological Sciences, University G. D’Annunzio Chieti-Pescara, Chieti, Italy

**Keywords:** Pelvic pain, Gynaecological examination, Ultrasonography, Computed tomography, Magnetic resonance imaging

## Abstract

**Abstract:**

Acute pelvic pain (APP) is a common presentation in women of all ages and has both gynaecological and non-gynaecological causes. In the emergency department, the suspected differential diagnosis dictates the chosen imaging modality. For premenopausal patients with APP, transabdominal ultrasound (TAUS) and transvaginal ultrasound (TVUS) are first-line investigations with high sensitivity and specificity for ectopic pregnancy, adnexal torsion, and ovarian cysts and their complications. US may also be valuable in pelvic inflammatory disease. When a non-gynaecological cause is suspected, contrast-enhanced CT (± transabdominal US) is indicated and has the advantage of 24/7 availability and lack of operator dependence. CT, however, may reveal an unexpected gynaecological cause of APP. When available, MRI is an excellent second test to improve diagnostic certainty in pregnant women when US is inconclusive—both for gynaecological and non-gynaecological conditions. MRI has a high diagnostic accuracy for pelvic inflammatory disease and tubo-ovarian abscesses. This article will enable readers to refresh their knowledge of common causes of APP and understand the histopathological processes involved in gynaecological causes of APP and how the imaging findings correlate. It will outline why different modalities are useful in different pathologies and help understand the limitations of each modality, including the requirement for operator expertise (US), relative lack of specificity/sensitivity (CT), and limited availability (MRI). This article excludes pregnancy-related causes of APP (apart from ectopic pregnancy) and also excludes non-gynaecological causes of APP.

**Key Points:**

- *In female patients with acute pelvic pain, ultrasound is the best first modality in suspected gynaecological pathology.*
- *CT can be used when non-gynaecological causes of pain are suspected and when US is inconclusive.*
- *MRI has limited availability in an emergency setting and may be used in pelvic inflammatory disease and in pregnancy when US is inconclusive.*

## Key recommendations

- In skilled hands, transabdominal and transvaginal ultrasound is the best first modality in suspected gynaecological pathology. It will help identify ectopic pregnancy, adnexal torsion, causes of ovarian cysts and their complications, and may be of value in pelvic inflammatory disease (Level of evidence high).
- CT is a relatively non-specific test which is often the first imaging investigation due to its easy availability 24/7. It can be used when non-gynaecological causes of pain are suspected and when US is non-conclusive (Level of evidence moderate).
- MRI has high diagnostic accuracy and is very helpful in further characterisation after US ± CT. It has limited availability in an emergency setting and may be used in differentiating pelvic inflammatory disease and tubo-ovarian abscesses. MRI is useful in pregnancy when US is inconclusive (Level of evidence moderate).

## Introduction

### Imaging modalities and protocols

Acute Pelvic Pain (APP) is a common emergency complaint (Table 1) with gynaecological and non-gynaecological causes in women of all ages [1]. The differential diagnosis determines the choice of imaging modality. Ultrasound (US) is indicated if a gynaecological cause is suspected and has many advantages, being non-invasive, radiation-free, and with a high diagnostic yield. Transabdominal (TA) and transvaginal (TV) US are often performed together. TAUS allows visualisation of all pelvic contents, including free pelvic fluid. TVUS better depicts the uterus and adnexa. Colour and spectral Doppler are useful for vascularity assessment, especially if torsion is suspected [2] (Table 2). However, CT is increasingly becoming the first emergency imaging test due to 24/7 availability, especially if a non-gynaecological cause of APP is suspected (such as appendicitis or renal colic) and when US is unavailable [2, 3].

**Table 1:** Causes of acute pain

Gynaecological		Non-gynaecological
Nonpregnant	Pregnant	Genitourinary
• Ovarian functional cyst rupture/haemorrhage	• Haemorrhagic corpus luteal cyst	• Distal ureteral calculus
• Pelvic inflammatory disease (PID)	• Ectopic pregnancy	• Lower urinary tract infection
• Ovarian torsion	• Uterine rupture	Gastrointestinal
• Fibroid degeneration/torsion	• Uterine torsion	• Appendicitis
• Malpositioned IUD	• Placental abnormalities	• Diverticulitis
• Hematometra	• Spontaneous/incomplete abortion	• Epiploic appendagitis/intraperitoneal focal fat infarction
• Endometriosis/endometriosis cyst rupture	• Ovarian hyperstimulation syndrome	• Bowel inflammation, ischaemia, haemorrhage
• Ovarian hyperstimulation syndrome		
• Gynaecological tumours		
• Ovarian vein thrombophlebitis

**Table 2:** US findings of common causes of acute pelvic pain

Pelvic inflammatory disease (PID)	• Free fluid in the pelvis with internal echoes indicating purulent content • Endometritis/pyometra: thickened heterogenous endometrium, indistinct endometrium, fluid and/or gas within the cavity • Salpingitis: swollen fallopian tube (> 5 mm diameter), thickened walls and endosalpingeal folds showing hyperaemia on Colour Doppler. • Pyosalpinx: dilated fallopian tubes with echogenic fluid that may form levels due to purulent content • Tubo-ovarian abscess: multilocular complex cystic mass in the adnexa with a thick wall and internal echoes, showing hyperaemia on Colour Doppler. The ovary and the fallopian tube cannot be individually identified. Gas may be seen as echogenic foci with posterior dirty shadowing.
Ovarian cysts	• Follicular cyst: thin wall, posterior acoustic enhancement. No internal vascularity on Colour Doppler • Corpus luteum: well-circumscribed cyst ≤ 3 cm with a thick wall showing prominent hyperaemia on Colour Doppler (“ring of fire” sign), and no internal vascularity. Spectral Doppler: prominent diastolic flow with low-velocity waveform throughout the luteal phase of the cycle. • Haemorrhagic cyst: heterogeneous content, fluid levels, possible complex mass appearance. No internal vascularity. ± Hemoperitoneum • Haemorrhagic corpus luteum: heterogeneous echogenic content, thickened hyperaemic walls. ± Hemoperitoneum
Adnexal torsion	• Enlarged ovary with peripherally displaced follicles. Heterogeneous ovarian echotexture (echogenic areas = haemorrhage; hypoechoic areas = oedema) • Midline or superior displacement of the affected ovary. Uterine deviation to the side of the twist. Ascites • Colour Doppler: Whirlpool sign indicating the twisted pedicle • Spectral Doppler: absent venous flow, decreased/absent diastolic flow, absent arterial flow. Important: presence of arterial or venous flow does not exclude ovarian torsion.
Ectopic pregnancy (serum β-hCG +)	• Absence of a normal intrauterine gestational sac (double decidual sac with two concentric hyperechoic rings that surround an anechoic gestational sac) • Tubal pregnancy: ∘ Adnexal mass separate from the ovary ± presence of gestational sac or a living embryo ∘ “Tubal ring sign” + “ring of fire sign”: decidual response in the fallopian tube with hypervascularity • Extrauterine findings: pelvic free fluid, hematosalpinx, hemoperitoneum. Hemoperitoneum is highly suggestive of ruptured ectopic pregnancy

CT and US in combination may improve diagnostic certainty in some patients [4]. A single venous phase minimises radiation, with an additional arterial phase warranted in suspected active bleeding [3, 5].

In dual-energy CT (DECT), iodine mapping can differentiate haemorrhagic infarction from contrast enhancement in ovarian torsion. Virtual monochromatic images increase the detection of ischaemia in adnexal torsion or peritoneal inflammation. Finally, DECT can replace a true nonenhanced scan with a virtual nonenhanced scan, reducing radiation exposure [6].

On CT, normal fallopian tubes are not visible, while normal ovaries can be identified by location and follicular structure [5].

MRI, if available, enables excellent characterisation of abnormal gynaecological findings already identified on US or CT without ionising radiation [5]. A full MRI protocol includes fat-suppressed T2W images which increase the conspicuity of inflammation, oedema, and ascites. Gradient echo T2* sequences and pre-contrast T1 fat-saturated sequences identify blood products. DWI detects hypercellular fluid/pus [7].

In APP, gadolinium-enhanced fat-suppressed T1W demonstrates inflammatory peritoneal enhancement in PID (not visible in endometriosis). It can also characterise leiomyomas, leiomyosarcomas and adnexal masses [5, 8] (Table 3, limited protocol).

**Table 3:** Time-efficient non-contrast MRI protocol for urgent study of the female pelvis and non-cooperating patients

MRI protocol	Axial T2W SSFSE	Sagittal T2WFRFSE	Oblique coronal/axial T2W FRSE	Axial DWI SE EPI	Sagittal, oblique coronal/axial T1W 3D GRE LAVA
Repetition time/echo time (ms)	765/59	4675/100	4675/100	3000/74	4.4/2.1
Flip angle	90°	90°	90°	90°	12°
Section thickness (mm)	6	4	4	8	3.4
Interslice gap (mm)	0.6	0.4	0.4	2	−1.7
Bandwidth (kHz)	31.25	41.67	41.67		62.5
Field of view (cm)	38	32	32	42	40
Matrix	320 × 288	320 × 224	320 × 224	160 × 160	370 × 192
No. of averages	0.54	2	2	2	0.75
No. of images	30	26	26	15	
Frequency direction	Right to left	Anterior to posterior	Right to left	Anterior to posterior	Superior to inferior
Acquisition time	24 s	2 min 15 s	2 min 15 s	27 s	22 s
β value (s/mm^2^)	-	-	-	0–800	-

Reproduced under the Creative Commons Attribution 4.0 International License (http://creativecommons.org/licenses/by/4.0/) from Tonolini et al [5]

*T2W* T2-weighted, *T1W* T1-weighted, *SSFSE* single-shot fast spin-echo, *FRFSE* fast recovery fast spin-echo, *DWI* diffusion-weighted imaging, *SE* spin-echo, *EPI* echoplanar imaging, *GRE* gradient echo, *LAVA* liver acquisition with volume acceleration

### Gynaecological causes of acute pelvic pain

#### Infection/pelvic inflammatory disease (PID)

PID refers to the inflammation of female reproductive organs, typically caused by bacterial infection ascending from the vagina, causing cervicitis, endometritis, salpingitis, pyosalpinx, oophoritis, tubo-ovarian abscess (TOA) [9], peritonitis and occasionally pyometra and ovarian vein thrombophlebitis [10]. Risk factors include multiple sexual partners, intrauterine surgery, intrauterine devices (IUDs), delivery, and endometriosis [7].

Fever, pelvic pain, vaginal discharge, cervical tenderness and dyspareunia are common presentations. PID requires early antibiotic treatment to avoid complications such as infertility and ectopic pregnancy [10, 11]. Imaging helps in clinical uncertainty. Ultrasound is the best modality with CT used if US is inconclusive or there are suspected complications. General CT findings include thickening of the uterosacral ligaments, pelvic fat stranding (sensitivity 60.4%), obscuration of fascial planes, reactive lymphadenopathy, and pelvic free fluid [10, 11]. The most specific CT finding is bilateral tubal thickening (95.1%, *n* = 190) [12].

MRI has a greater sensitivity than CT (0.95 pooled sensitivity versus 0.79) [13]. However, MRI is rarely needed to diagnose PID except in doubtful cases, the differential diagnosis of unclear adnexal lesions, and distinguishing non-complex fluid from blood and pus [7, 14].

##### Cervicitis and endometritis

Both represent early manifestations of PID. In cervicitis, the cervix may be enlarged and hyperaemic on US, with an enhancing endocervical canal and parametrial fat stranding and free fluid on CT/MRI. Endometritis can occur in PID, during the peripartum period, and after gynaecological procedures. Findings of a thickened heterogenous endometrium on US, abnormal endometrial enhancement relative to the inner myometrium on CT/MR, and fluid within the cavity suggest endometritis. The uterine border may be indistinct from parametrial tissue [2, 10]. Uterine empyema (pyometra) is characterised by complex fluid in the uterine cavity containing gas or air-fluid levels [10].

##### Salpingitis, tubal empyema (pyosalpinx)

**Fig. 1:**
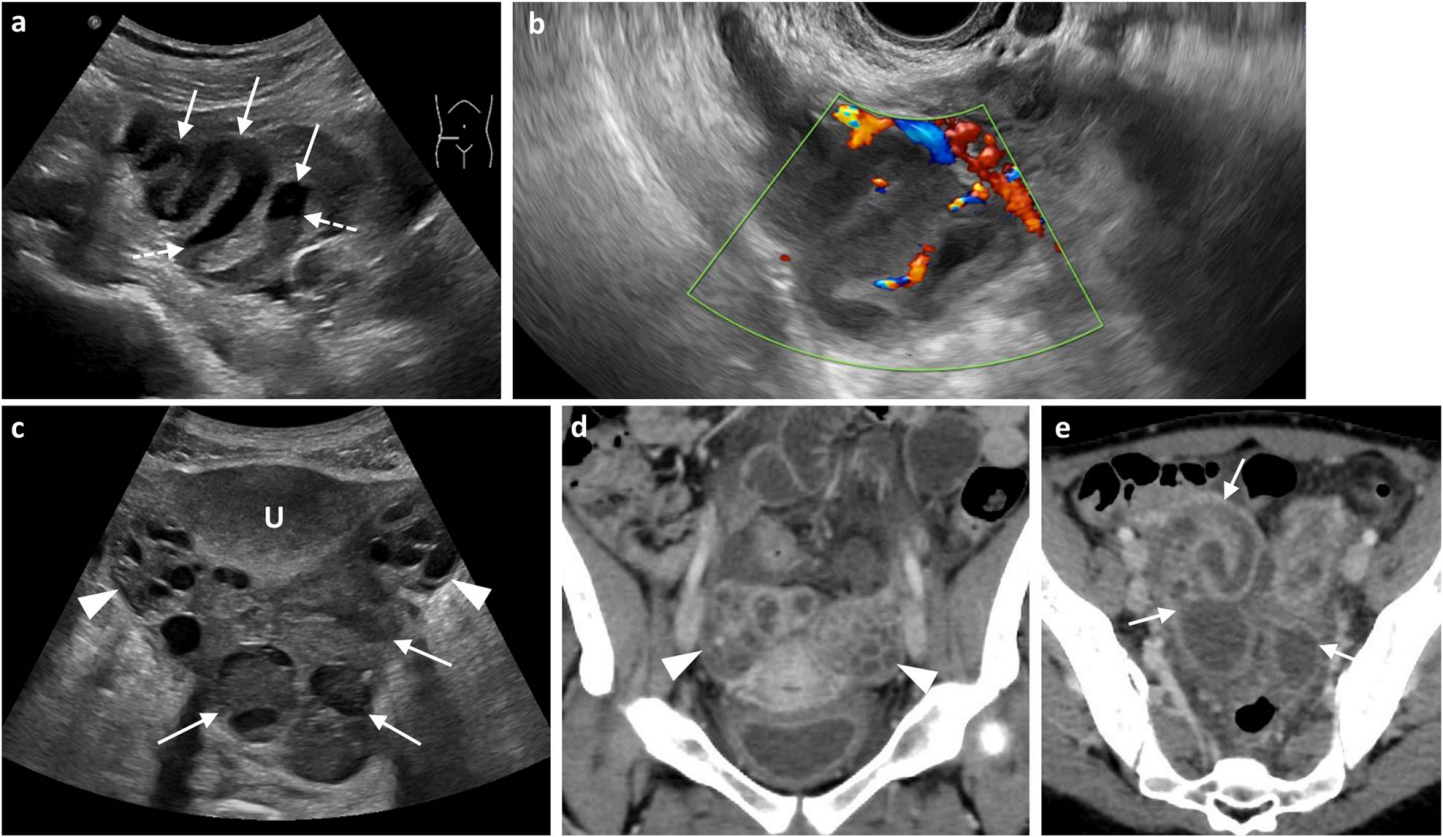
Pelvic inflammatory disease (pyosalpinx) in two different patients presenting with acute pelvic pain and fever. **a** Transabdominal grey-scale US and (**b**) transvaginal Doppler image show thick-walled dilated tubular adnexal structures (arrows in **a**), distended with echogenic fluid-debris levels (dashed arrows) with surrounding vascularity with Doppler ultrasound. **c** Transabdominal grey-scale US image in a different patient, shows bilateral cystic structures (arrows) surrounding the uterus (U) containing hyperechoic material compatible with pyosalpinx. The ovaries show multiple follicles (arrowheads). **d**, **e** Coronal and axial post-contrast CT images, respectively, of the same patient, demonstrate both ovaries with multiple follicles (arrowheads) and bilateral thick-walled dilated tubular structures (arrows) representing the dilated fallopian tubes

In salpingitis, the fallopian tube is swollen (> 5 mm diameter) and thickened. In pyosalpinx, pus distends the lumen (echogenic on US) and the mural thickening is hyperaemic on Doppler US, enhancing on CT (Fig. 1). CT may show surrounding pelvic inflammation, including uterosacral ligament thickening and para-aortic lymphadenopathy [2, 10, 15]. CT-multiplanar reconstruction helps identify the tubular contour, while MRI additionally differentiates pyosalpinx from haematosalpinx by the presence of intratubal blood products. In pyosalpinx, there is tubal wall thickening, enhancement and inflammation. On DWI, restricted diffusion suggests pyosalpinx, while unrestricted diffusion suggests hydrosalpinx [7].

##### Tubo‑ovarian abscess

**Fig. 2:**
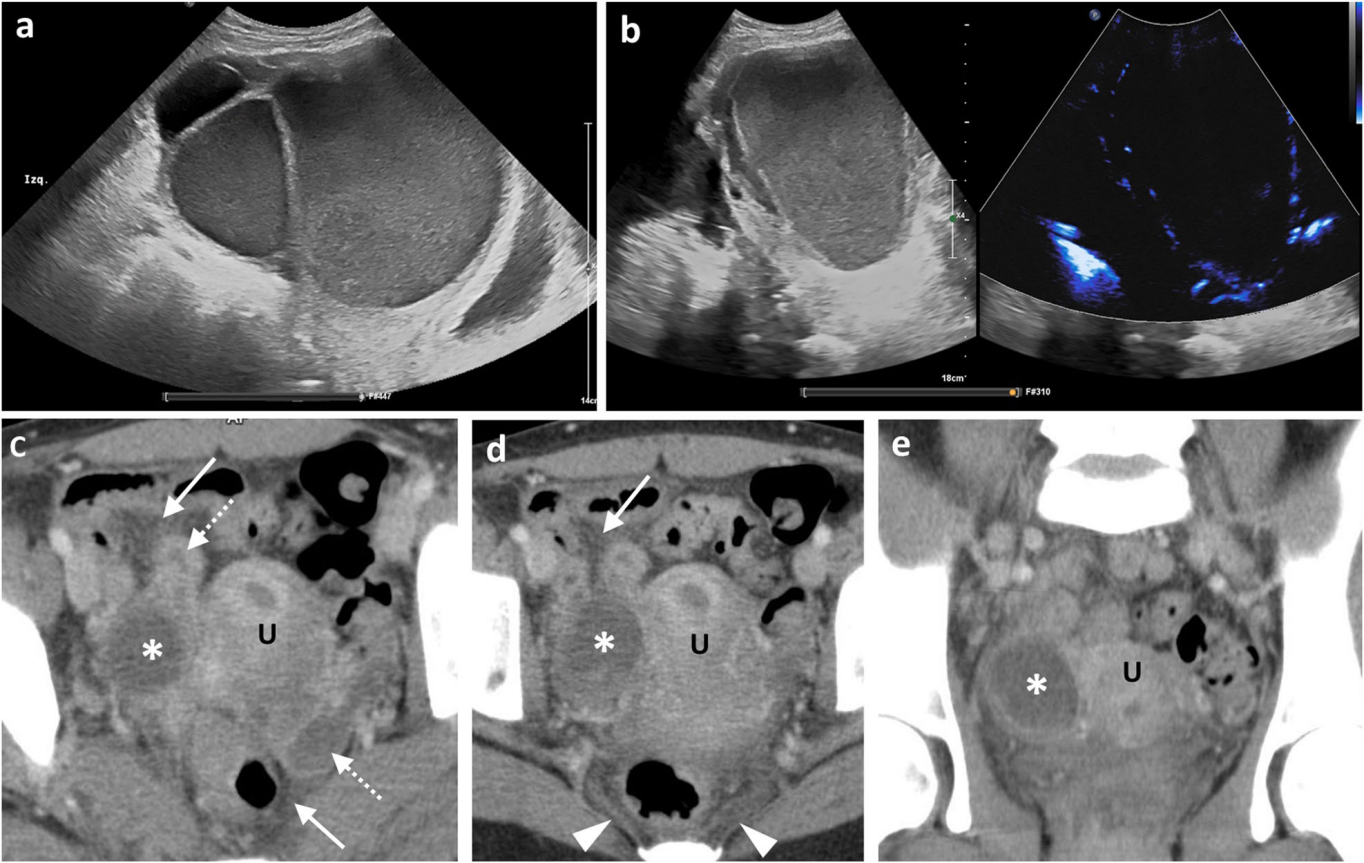
Surgically confirmed tubo-ovarian abscess in two different patients. **a**, **b** Middle-aged woman with lower abdominal pain, fever and vaginal discharge. **a** Transabdominal grey-scale ultrasound shows a large cystic complex mass representing a tubo-ovarian complex with echogenic content consistent with pus. **b** Microflow ultrasound imaging demonstrates peripheral vascularity in the walls of the cystic lesion without internal vascularity in keeping with the cystic nature of the mass. **c**–**e** Young woman with pelvic pain and raised inflammatory markers. Consecutive axial (**a**, **b**) and coronal (**c**) CECT images, venous phase, show bilateral pyosalpinx (dotted arrows) and a right ovarian abscess (asterisk). Note the fat stranding around the fallopian tubes (arrows) and the markedly thickened uterosacral ligaments (arrowheads)

Infection and destruction of adnexal structures result in a TOA. Imaging findings typically reveal a multilocular complex cystic mass in the adnexa with thick walls, showing hyperaemia on US and uniform enhancement on both CT and MRI (Fig. 2). Associated free peritoneal fluid, surrounding pelvic inflammation, fat stranding and enhancement are common [10, 15]. The complex mass can be difficult to differentiate from an ovarian malignancy, but a dilated fallopian tube and restricted diffusion on DWI sequences suggest infection [11].

##### Peritonitis

Pyometra, pyosalpinx, and TOA can cause peritonitis when an abscess ruptures or pus leaks from the infected organ [10, 15], but it can also be seen in the absence of such complications.

##### Fitz-Hugh–Curtis syndrome (perihepatitis)

In this rare complication, peritoneal spread to the liver capsule results in sharp right upper quadrant (RUQ) pain. CT findings include enhancement and thickening of the anterior liver capsule, geographic areas of variable perfusion in subcapsular and periportal areas, fluid and fat stranding extending from the pelvis into the RUQ, and gallbladder wall thickening [10, 15].

##### Atypical PID forms

PID can uncommonly progress to involve other pelvic organs such as the bladder, urethra, or bowel. In addition to typical organisms, two further organisms should be considered, especially in unusual or extensive cases: Actinomycosis is suggested when there is extension across tissue planes and fistula formation in the presence of an IUD. Tuberculosis can cause pyosalpinx, endometritis, and peritoneal thickening with deposits that can mimic peritoneal carcinomatosis [15].

##### Vaginal infections

Isolated vaginal abscesses (without ascending infection) can occur in Bartholin’s glands (posterolateral vagina) and in a Gartner cyst (anterolaterally within the proximal vagina) [11].

#### Uterine emergencies: leiomyoma degeneration and uterine inversion

Uterine leiomyomas (fibroids) are a common gynaecological neoplasm containing smooth muscle and fibrous connective tissue, which can be submucosal, intramural, subserosal, and/or pedunculated [11, 16]. In 30% of patients, acute degeneration or torsion can cause APP [16]. Ultrasound is helpful, but MRI is optimal in characterisation [16].

**Fig. 3:**
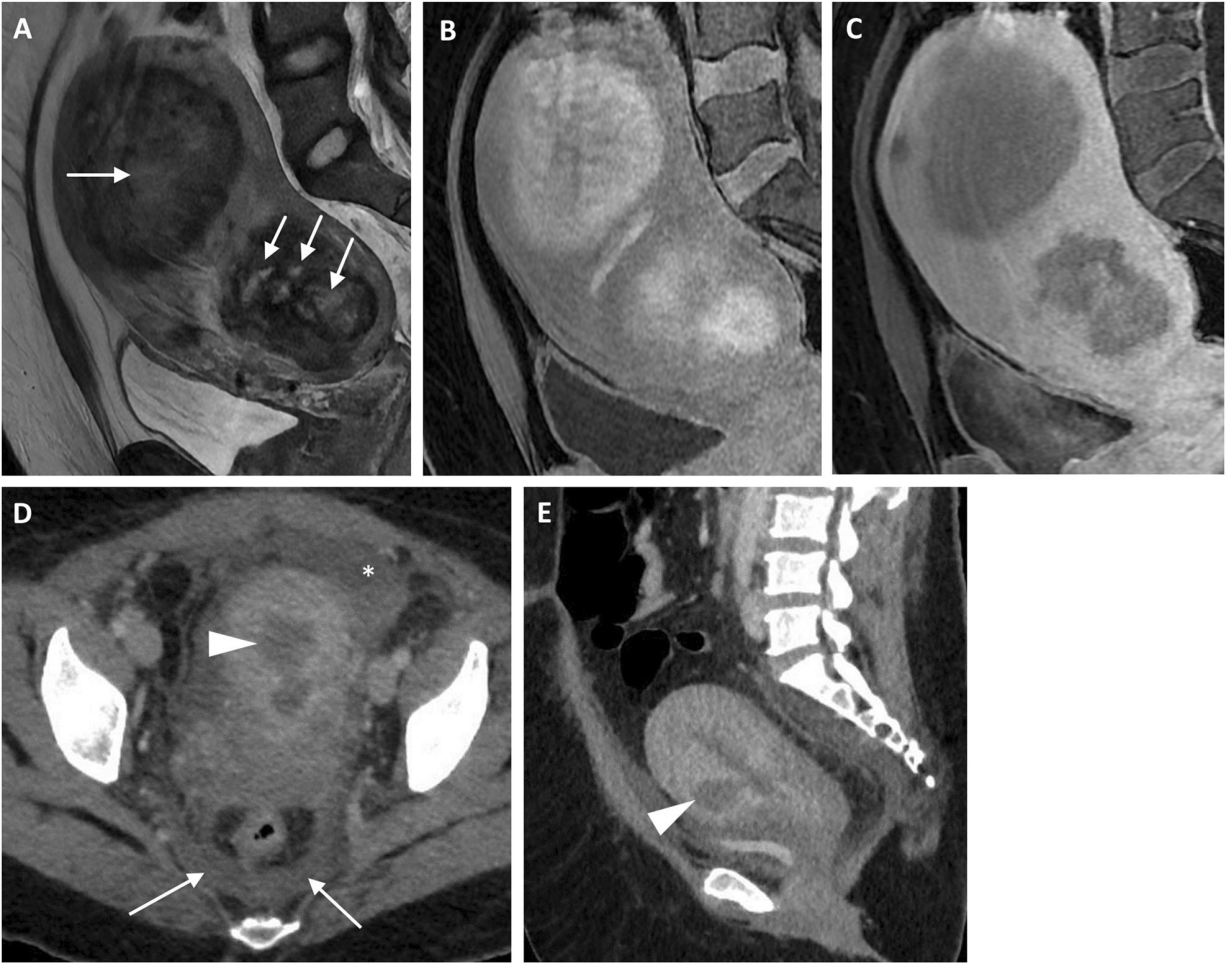
**A**–**C** Red degeneration on an intramural fibroid in a postmenopausal patient with acute pelvic pain and known fibroids. Sagittal T2, T1-FS pre and post-gadolinium MRI images, respectively, show intramural fibroids (arrows) with (**A**) central heterogeneous T2 signal, (**B**) hyperintense on T1 fat sat (haemorrhage) and (**C**) non-enhancement in the post-contrast images in keeping with infarction. **D**, **E** Myometrial abscess in a patient with pelvic pain and vaginal discharge. Axial and sagittal post-contrast CT images show pelvic fluid (asterisk), fat stranding and thickening and oedema of the uterosacral ligaments (arrows). A uterine intramural hypodense abscess (arrowheads) was noted communicating with the endometrial cavity which is distended with fluid

In acute degeneration, the leiomyoma outgrows its blood supply. Speed of onset of degeneration influences pain experienced, histopathological nature (hyaline, myxoid, cystic or haemorrhagic) and imaging findings [16]. Hyaline degeneration is the most common and least painful, with deposition of collagen fibres [11]. Haemorrhagic (so-called ‘red’) degeneration occurs with rapid leiomyoma growth, such as in pregnancy or with oral contraceptive use [16]. In red degeneration, CT may show haemorrhage, loss of contrast enhancement, ± cystic contents [11]. On MRI, non-degenerated leiomyomas are typically well-circumscribed, low to intermediate signal intensity on T2. Degenerated leiomyomas do not enhance post gadolinium due to infarction and, on T2W imaging, can be low (hyaline or calcific degeneration) or high signal (cystic or myxoid degeneration) [16]. Red degeneration may show peripheral T1 high signal intensity (representing blood products and possibly thrombosed peripheral vessels) with variable T2 signal intensity (Fig. 3).

Of patients who have undergone uterine artery embolisation of leiomyomas, 10% may require readmission for postembolisation syndrome, which includes pain, fever, and nausea. MRI will demonstrate haemorrhagic infarction (high signal on T1) and lack of enhancement correlating with successful embolisation [16].

Inversion of the uterine fundus is rare, can extend through the cervix, and occurs either acutely postpartum or in postmenopausal multiparous women, where it may be associated with a leiomyoma acting as a lead point [11]. While this is a challenging clinical diagnosis, sagittal MRI (and, to a lesser extent, CT) demonstrates inversion and indentation of the fundus [17].

#### Ovarian cyst complications, endometriomas, and ovarian hyperstimulation syndrome

##### Haemorrhagic and ruptured ovarian cysts

**Fig. 4:**
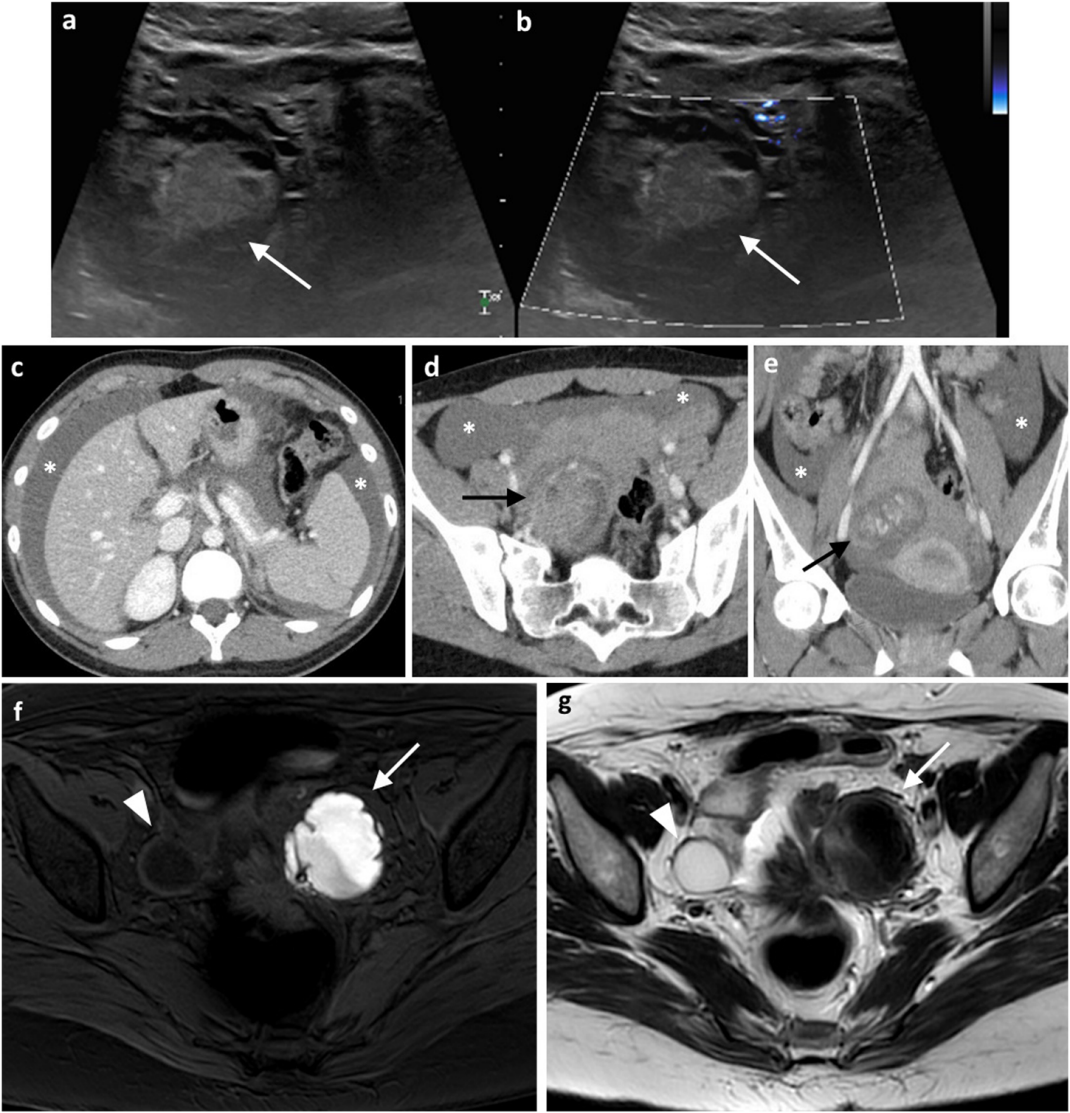
Complicated ovarian cyst in a young woman in her late teens presenting with acute pelvic pain, nausea and vomiting. **a**, **b** Transabdominal grey-scale and Doppler US shows a complex cystic mass with hyperechoic content (arrow) without vascularisation in Doppler images. **c**–**e** Portal venous phase axial and coronal CT images demonstrate a large amount of hemoperitoneum (asterisk) in the abdomen and pelvis. The images through the pelvis show a right para-uterine cystic mass (black arrows) with multiple hyperdense foci within related to active bleeding. Large hemoperitoneum with sentinel clot in the pelvis. Findings suspicious for ruptured ovarian cyst with active bleeding. Surgery confirmed ruptured corpus luteal cyst. **f**, **g** Endometrioma in a young woman with acute left quadrant pain. Axial T1-FS WI (**f**) shows a large left ovarian cyst with high signal intensity and a fluid-fluid level (arrow). In the axial T2 WI (**g**), the adnexal lesion shows marked signal loss due to cycling bleeding (shadowing sign). Note the right ovarian follicular cyst (arrowhead)

Ruptured or haemorrhagic ovarian cysts are the most common gynaecological cause of APP in a nonpregnant, afebrile premenopausal woman [18]. This may be a physiologic, self-limited process involving a corpus luteum (CL) cyst or a follicular cyst [6] (Fig. 4). While ovarian endometriosis is common, rupture of an endometriotic cyst is uncommon [19].

Ruptured or haemorrhagic ovarian cysts present clinically with severe APP and, occasionally, hypotension due to large intraperitoneal bleed [18]. B-hCG levels help to differentiate from ruptured ectopic pregnancy [14].

US is the best modality to assess ovarian cyst rupture and haemorrhage/haemoperitoneum. Ruptured ovarian cyst may be sonographically normal if the cyst has completely ruptured and the fluid reabsorbed. Clues to a leaking cyst are crenellated appearance and low-level echoes/clot. Ruptured luteal cysts have a thick, echogenic, and irregular wall with increased peripheral blood flow on Doppler, the so-called “ring of fire” [14, 18].

Sonographic findings of haemorrhagic cysts depend on the age of the haemorrhage. In the early stages, a haemorrhagic cyst exhibits diffuse, low-level internal echoes, thin walls, posterior acoustic enhancement and no internal vascularity (Fig. 4) [18]. As the haemorrhage evolves, a lace-like reticular pattern of internal echoes develops. As echogenic thrombus coalesces within the cyst, a heterogeneous avascular mass forms with retractile angular or concave margins. Clots may be adherent or rounded but can be differentiated from mural nodules by lack of vascularity on Doppler US and, where needed, 6–8 week US follow-up [18]. On CT, high-density cyst contents and thick enhancing walls can be seen. The MR appearance of haemorrhagic cysts is complex, with different signal characteristics at different stages [6].

##### Complicated endometriomas

Endometriosis is the presence of ectopic endometrial tissue outside of the uterus. In the ovary, ectopic endometrial tissue can form haemorrhagic cysts or endometriomas due to repeated cyclic haemorrhage causing APP [5]. On US, endometriomas are unilocular, homogeneously hypoechoic cysts with diffuse low-level echoes (‘chocolate cysts’) [18]. Spontaneous rupture of an endometriotic cyst is rare and torsion rarely occurs because of adhesions, however, these should still be considered in patients with endometriosis. CT can demonstrate features suggestive of endometrioma rupture, including thick-walled, multilocular or bilateral ovarian cysts, loculated pelvic ascites, and fat stranding [19].

Other acute complications of endometriosis causing APP include endometrioma superinfection, PID, hemoperitoneum, and bowel and genitourinary complications [20].

MRI is the best technique to diagnose endometriomas with ‘shading’ on T2W due to dependant blood and repeated bleeding episodes (Fig. 4), with bilateral or multiple lesions being typical. In comparison, a non-endometriotic haemorrhagic ovarian cyst is more likely to be unilateral, unilocular, and without shading.

##### Ovarian hyperstimulation syndrome

**Fig. 5:**
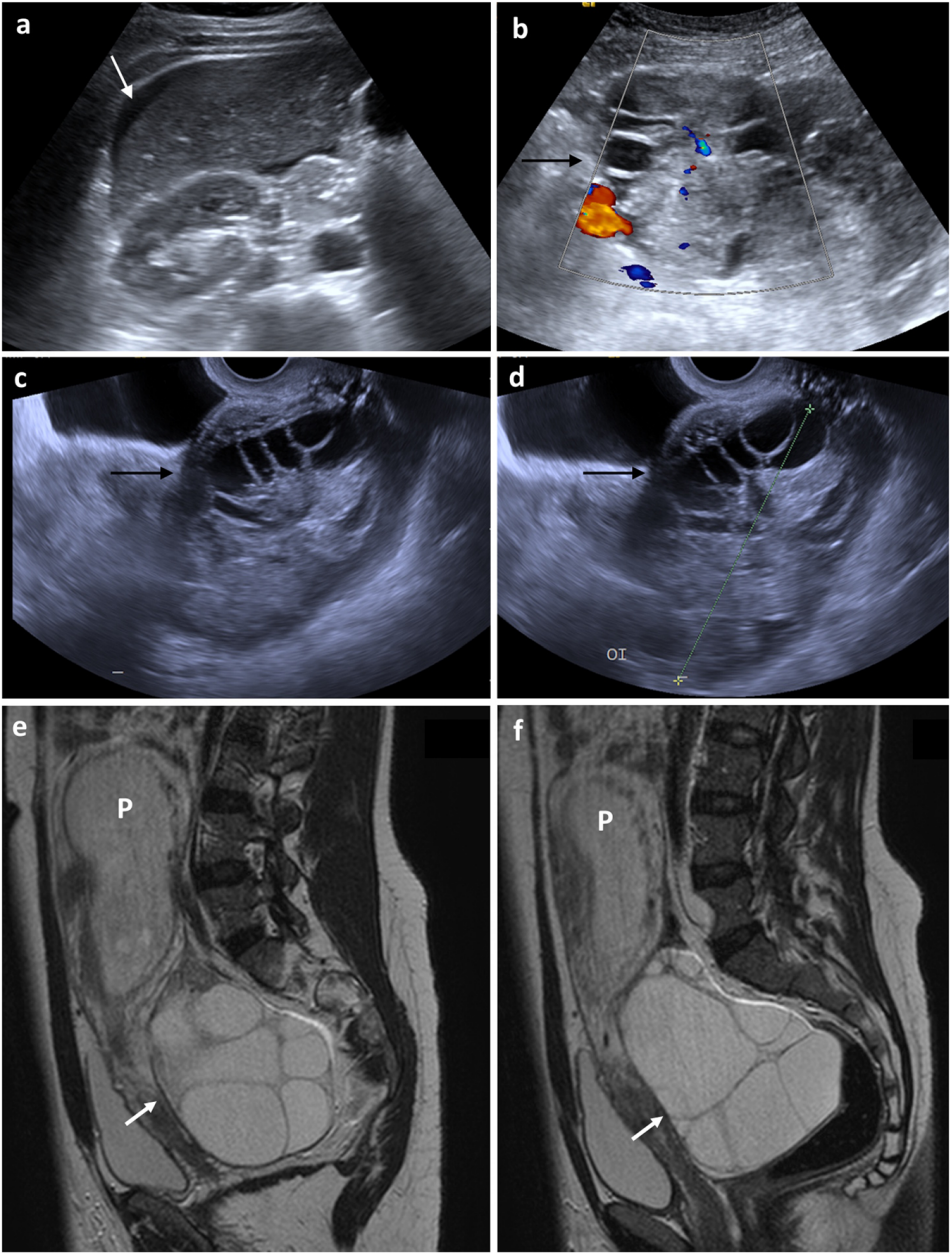
Ovarian hyperstimulation syndrome in two different patients. **a**–**d** Women in her 30’s undergoing in vitro fertilisation presented with abdominal distention, nausea and vomiting. TAUS (**a**, **b**) and TVUS (**c**, **d**) demonstrate ascites (arrow in **a**), enlarged ovaries (black arrows) with preserved central flow (**b**) and multiple follicles of varying sizes (**b**–**d**) consistent with OHSS. **e**, **f** Different patient in her early 30’s and 15 weeks pregnant presented with abdominal pain. Sagittal T2WI at the level of the right and left adnexal regions show markedly enlarged bilateral ovaries (arrows) containing multiple large, thin-walled cysts consisted with OHSS. P (placenta). MRI can provide better characterisation of the ovarian process in the differential diagnosis of ovarian tumours

Ovarian hyperstimulation syndrome (OHSS) is a condition that can occur after ovarian stimulation, often in the context of fertility treatments. Imaging plays a crucial role in diagnosing and assessing the severity of OHSS. Ultrasound is the primary imaging modality used to evaluate the ovaries, where findings typically include enlarged ovaries with multiple thin-walled cysts, ranging from small to large, representing enlarged follicles or corpus luteum cysts (Fig. 5). In severe cases, free fluid may be seen in the pelvis or abdomen due to capillary leakage. In more advanced stages, CT or MRI scans may be employed to assess complications such as ovarian torsion, ascites, pleural effusions or other signs of severe OHSS. Particularly, MRI is very helpful in differentiating OHSS from ovarian tumours, including choriocarcinoma, which can also produce high hCG levels, by demonstrating markedly bilateral symmetrically enlarged ovaries with simple cysts of different size, separated by thin septa and lacking inhomogeneous solid tissue consistent with malignancy (Fig. 5) [5].

#### Adnexal torsion

Adnexal torsion can involve the ovary, fallopian tube, or (most commonly) both. Isolated fallopian tube torsion spares the ovary, is rare, usually occurs during reproductive years and seldom post-menopause [6]. Clinically, patients present with nausea, vomiting and excruciating unilateral APP [18].

A normal ovary rarely undergoes torsion, except occasionally in perimenarchal girls or pregnancy [6]. In up to 90% of cases, an underlying ovarian mass serves as the lead point for torsion, particularly if larger than 5 cm [14, 21]. Malignant ovarian tumours and endometriotic cysts are less likely to cause ovarian torsion due to adhesions [6]. Other risk factors include ovulation induction, pregnancy, previous tubal ligation and hypermobility of adnexal structures [18].

Ovarian torsion is a surgical emergency. Delayed treatment increases the risk of vascular compromise and haemorrhagic infarction. Torsion occurs when the ovary twists on its vascular pedicle, resulting in partial to complete obstruction of arterial inflow and venous outflow [22].

**Fig. 6:**
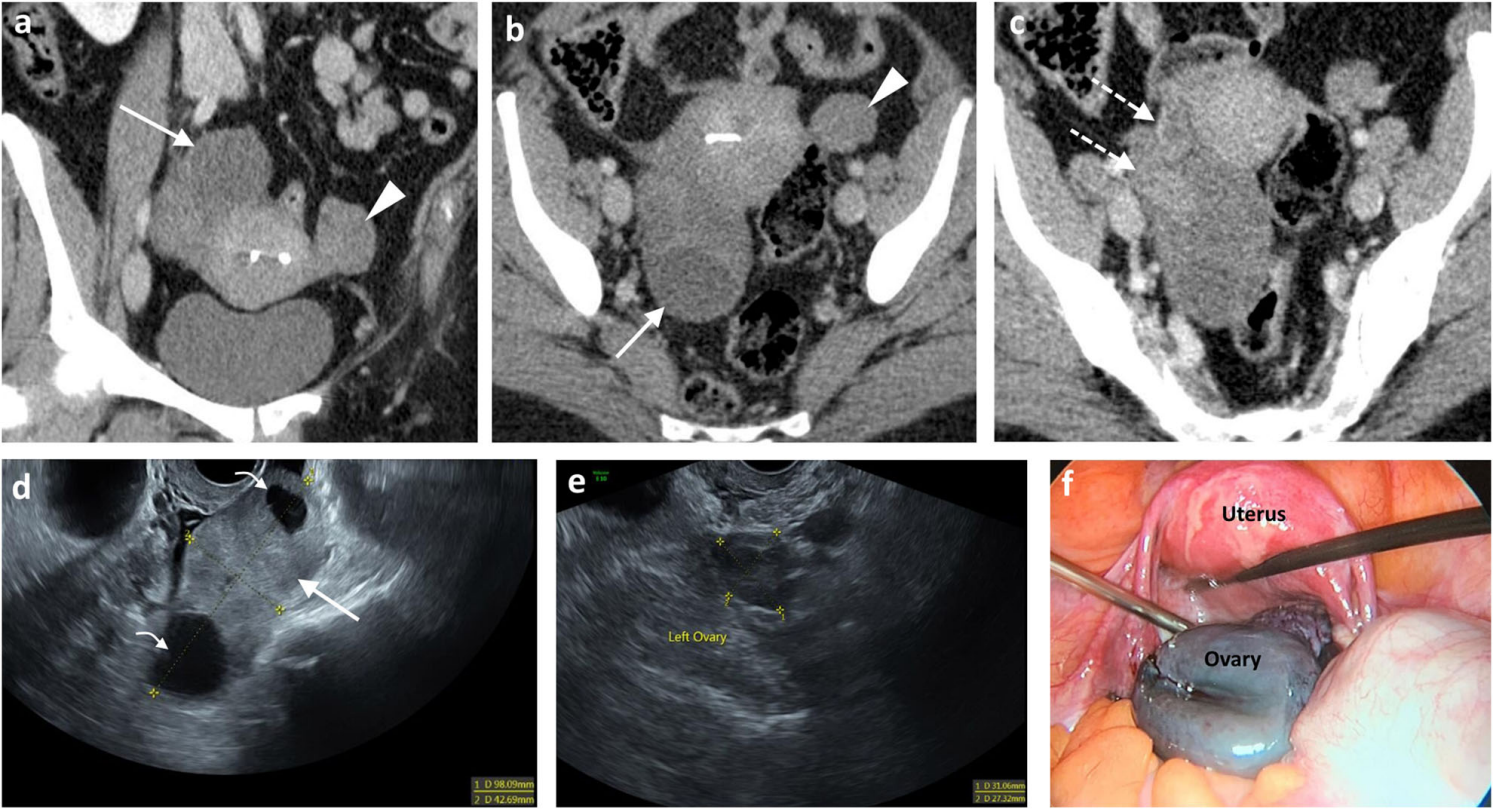
Right adnexal torsion in a woman in her 40’s with intense right pelvic pain and vomiting. CT was requested to exclude appendicitis. **a**–**c** Post-contrast coronal (**a**) and axial CT images (**b**, **c**) excluded appendicitis and showed an enlarged and hypodense right ovary (arrow) posteriorly displaced behind the uterus in the axial images, compared to the normal size and located left ovary (arrowhead) and twisted right fallopian tube (dotted arrows). **d**, **e** Subsequent transvaginal US performed preoperatively by the gynaecologist confirmed the CT findings with an enlarged and oedematous right ovary (arrow) measuring approximately 98 × 42 mm with distended peripheral follicles (curved arrows). **f** Intraoperative image confirmed right ovarian necrosis

Pelvic US and colour Doppler is the first imaging modality to rule in/out adnexal torsion. The US appearance of torsion is variable and depends on the chronicity and degree of torsion, and whether there is some preservation of arterial flow to the ovary from its dual supply from the ovarian and uterine arteries [23, 24]. Findings greatly depend on factors such as duration and degree of vascular obstruction and whether it is intermittent [23]. Classical sonographic signs of ovarian torsion include increased ovarian diameter (> 4 cm) and volume (> 20 cm^3^ premenopausal or > 10 cm^3^ postmenopausal); however, in 5% of patients, no ovarian enlargement occurs [21]. Other signs of ovarian torsion are peripherally displaced follicles, midline or superior displacement of the affected ovary, heterogeneity of the central stroma with echogenic areas indicating haemorrhage and hypoechoic areas representing oedema, and uterine deviation to the side of the affected ovary and ascites [14, 18]. Doppler ultrasound flow patterns reflect the degree of vascular compromise and duration of torsion with venous flow affected before the high-pressure arterial flow. Signs of complete torsion include absent venous flow, decreased or absent diastolic flow and absent arterial flow. In partial torsion, arterial flow with high resistive spectral signal may be seen, In some cases of nonviable or complete torsion, peripheral arterial flow can be preserved, which may be due to the dual arterial supply and relative preservation of flow within the uterine artery [21, 23]. The ‘whirlpool’ sign refers to the twisted ovarian pedicle, and in association with an enlarged ovary, it is diagnostic of ovarian torsion [21]. Many of these findings can also be seen with contrast-enhanced CT and MRI, including the presence of a twisted vascular pedicle or an underlying mass and abnormal enhancement (Fig. 6). Finally, abnormal morphology and cystic degeneration suggest infarction [21].

#### Haemoperitoneum due to gynaecological causes

Gynaecological causes of haemoperitoneum are many and include sexual intercourse, intense exercise, corpus luteum bleeding, ruptured haemorrhagic ovarian cyst, ectopic pregnancy, and ruptured endometriotic cyst. On US, peritoneal or pelvic fluid demonstrates low-level echoes, and US can also identify the cause. On CT, the free peritoneal fluid has a relatively high attenuation. CT can demonstrate the volume of haemoperitoneum, presence of septations or loculation (which suggest endometriosis), and active bleeding, which guides interventional management. Active bleeding may be seen on the arterial or, most usually, on the venous phase, reflecting intermittent or venous haemorrhage [5]. On MRI, signal intensity of haemoperitoneum depends on age of the blood [25].

#### Ectopic pregnancy including ruptured ectopic pregnancy

Ectopic pregnancy (EP) is a common cause of pregnancy-related APP. Early diagnosis and treatment have decreased the incidence of EP rupture, which is potentially life-threatening. In EP, the fertilised oocyte implants outside of the uterine endometrium, most commonly in the fallopian tubes (93–98% of all EP, of which 75% are ampullary, 13% isthmic and 12% fimbrial) [26]. The differential diagnosis includes non-gynaecological adnexal masses [27]. Table 4 outlines factors which increase the risk of EP.

**Table 4:** Factors which increase the risk of ectopic pregnancy [27]

Factors which increase the risk of ectopic pregnancy [27]
Previous PID
Previous surgery
Endometriosis
Use of IUDs
Previous EP
Assisted reproductive technology
Infertility
Smoking
Congenital uterine anomalies
Advanced maternal age

**Fig. 7:**
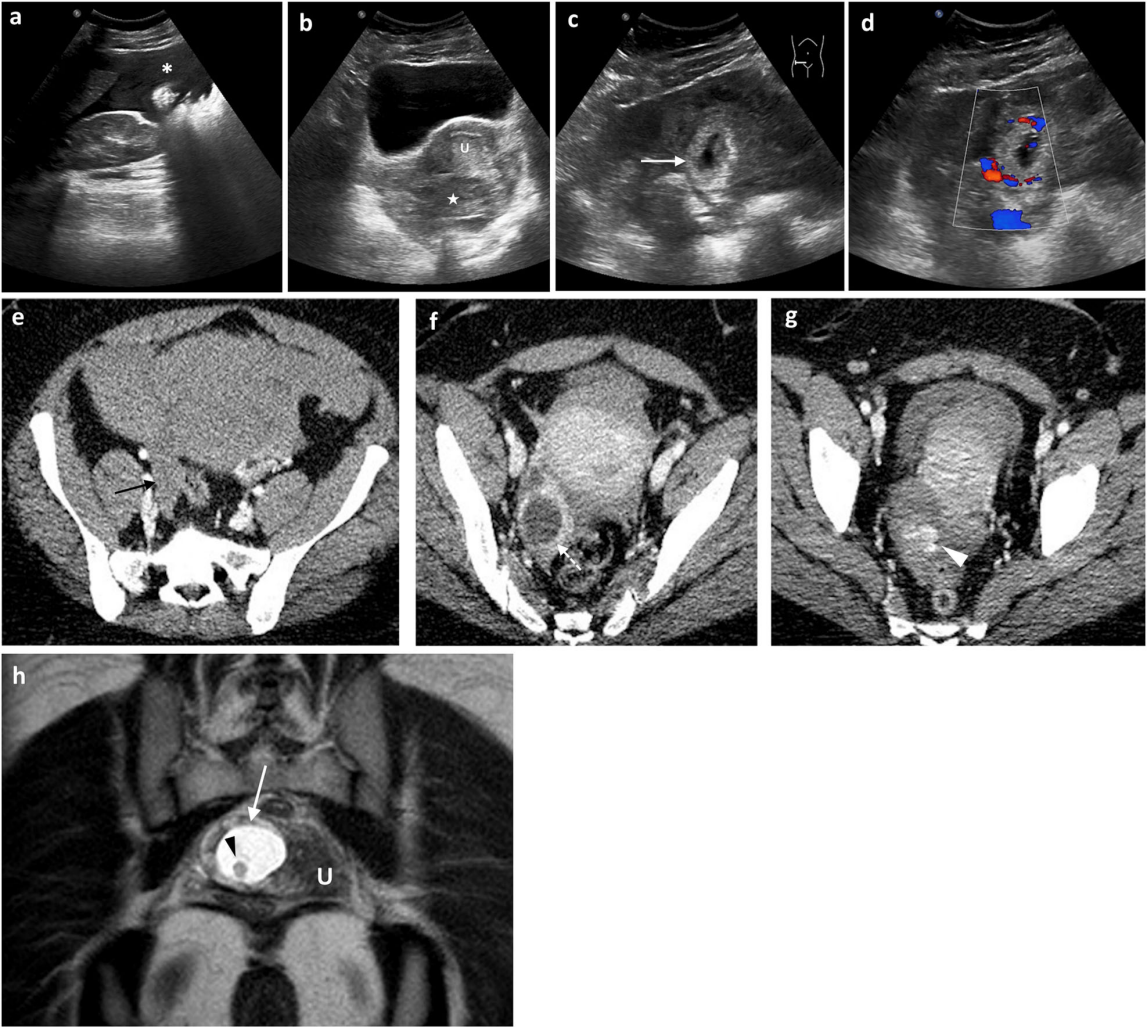
Ectopic pregnancies in three different patients. **a**–**c** Teenager with intense abdominal pain and persistent vaginal bleeding. Grey-scale US images show a large amount of partially echogenic abdominal fluid (asterisk) and a pelvic haematoma (star) surrounding the uterus (U). A round, thick-walled para-uterine cystic mass (arrow) with vascularised wall and a fetal pole was noted with Colour Doppler (**d**). No gestational sac was identified within the endometrial cavity. Laparoscopic surgery confirmed a ruptured ectopic tubal pregnancy with extensive hemoperitoneum. **e**–**g** Axial post-contrast CT images in a different patient with a ruptured tubal EP. Normal right adnexa is noted (black arrow), separate from a ring-enhancing right para-uterine cystic mass (dotted arrow) which increases the likelihood that the cystic mass represents an ectopic pregnancy. Active bleeding from the ruptured tubal pregnancy is visible during the arterial phase (arrowhead). **h** Woman in her 20’s presenting in the emergency department with acute pelvic pain and vaginal bleeding. Coronal T2 WI shows an eccentric gestational sac (arrow) with a fetal pole (arrowhead) located in the interstitial segment of the right Fallopian tube, next to the uterus (U). MRI is more helpful in evaluating ectopic interstitial pregnancy, which is a diagnostic challenge on ultrasound

A serum β-hCG value > 2000 mlU/mL (IRP International Reference Preparation*)* without intrauterine pregnancy but with an extraovarian mass is highly suggestive of an EP [28]. On TVUS, an adnexal mass separate from the ovary is seen in most, but not all, tubal pregnancies [29]. Other signs include the “tubal ring sign”: a thick echogenic ring surrounding an extrauterine gestational sac and the “ring of fire sign” due to peripheral hypervascularity of the hyperechoic ring (Fig. 7). If the trophoblast invasively grows into the fallopian tube, EP rupture and hemoperitoneum occurs.

Haemoperitoneum in EP is not necessarily indicative of tubal rupture but the larger the amount of fluid, the higher the likelihood of such.

CT is now increasingly performed on pregnant women either because pregnancy status is unknown, there is clinical deterioration before serum β-hCG is available, or the early urine pregnancy test is false-negative. On CT, an adnexal area of low attenuation with a dramatic enhanced ring adjacent to the ipsilateral ovary and associated haemoperitoneum suggests EP. In EP rupture, active bleeding can be seen (Fig. 7). The main CT differential diagnosis of EP is a CL cyst because (1) the wall of a CL may show strong enhancement, (2) a CL cyst may rupture in the peritoneum, and (3) a CL cyst may occur in the setting of early pregnancy. If clinical symptoms and serum β-hCG levels do not allow differentiation of these two entities, the site of the adnexal cystic mass may be a clue: a CL cyst is intraovarian, unlike an EP.

MRI is a second-line test in suspected EP, demonstrating haemoperitoneum, a heterogeneous, partly haemorrhagic adnexal mass representing the gestational sac and haematosalpinx within a dilated tube and mural enhancement [5]. MRI is better than CT at demonstrating direct signs (ectopic gestational sac—92% diagnostic accuracy) (Fig. 7) and indirect signs (haematosalpinx, adnexal haematoma, hemoperitoneum). 100% diagnostic accuracy is achieved if the gestational sac is visible along with two indirect signs [30]. MRI protocol should include T2*W sequences in 3 planes to identify low signal fresh haematoma within the adnexal mass with a sensitivity, specificity and accuracy of 95%, 100% and 96%, respectively, in diagnosing EP [31].

## Summary statement

**Fig. 8:**
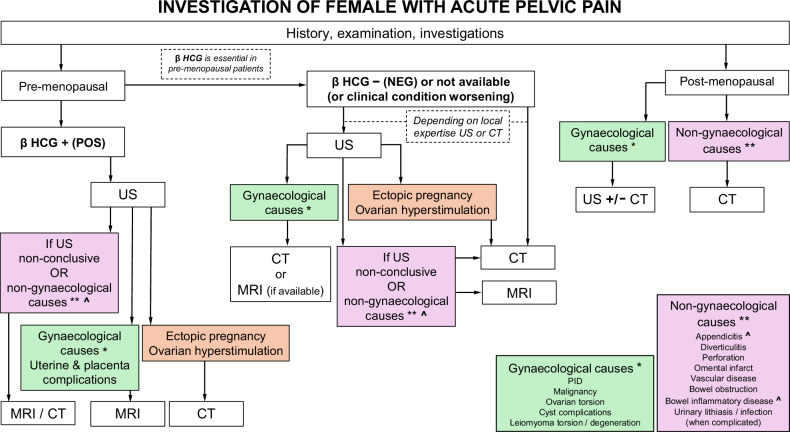
Flowchart of investigation of a female with acute pelvic pain. β HCG, beta humanchorionic gonadotropin; POS, positive; NEG, negative; US, ultrasound; CT, computed tomography; MRI, magnetic resonance imaging; PID, pelvic inflammatory disease. *Gynaecological causes, **Non-gynaecological causes, and ^^^MRI: consider MRI in these clinical scenarios in pregnant or very young women

Both gynaecological and non-gynaecological pathologies can cause acute pelvic pain, and both the clinician and radiologist have to bear this in mind when requesting and interpreting imaging. Pregnancy and pre- and postmenopausal status will direct investigation pathways Fig. 8. Common gynaecological causes include ovarian cyst haemorrhage, corpus luteum rupture, endometriomas, adnexal torsion, ectopic pregnancy, uterine leiomyoma degeneration, and a spectrum of infective pelvic inflammatory disease. Although ultrasound followed by MRI is often the preferred imaging pathway, the ubiquity of CT and the possibility of non-gynaecological causes means that for many patients with gynaecological causes of acute pelvic pain, CT is the first imaging modality.

## Patient summary

Acute pelvic pain in women can have both gynaecological and non-gynaecological causes. Gynaecological causes include corpus luteum rupture, ectopic pregnancy, twisting/loss of blood supply to fibroids or ovary, and infection of the cervix, uterus, fallopian tubes, and ovary. Ultrasound is the best first test if there is high suspicion of a gynaecological cause, whereas CT is often performed first if other causes, such as appendicitis or kidney stones, are suspected. MRI is usually a second-line test which confirms the suspected diagnoses seen on ultrasound or CT.

## Abbreviations

APP: Acute pelvic pain
CT: Computed tomography
DECT: Dual-energy CT
DWI: Diffusion-weighted imaging
EP: Ectopic pregnancy
IUD: Intrauterine device
MRI: Magnetic resonance imaging
RUQ: Right upper quadrant
TAUS: Transabdominal ultrasound
TOA: Tubo-ovarian abscess
TVUS: Transvaginal ultrasound
US: Ultrasound
β-hCG: Beta human chorionic gonadotropin
